# Appetite Loss in Metastatic Cancer: Insights from Clinical and Inflammatory Markers Observed Longitudinally

**DOI:** 10.3390/cancers18203407

**Published:** 2026-10-21

**Authors:** Ragnhild Habberstad, Ola Magne Vagnildhaug, Trude Rakel Balstad, Øyvind Salvesen, Erik Torbjørn Løhre, Asta Bye, Olav Dajani, Nina Aass, Stein Kaasa, Pål Klepstad, Tora S. Solheim

**Affiliations:** 1 Department of Clinical and Molecular Medicine, NTNU- Norwegian University of Science and Technology, 7491 Trondheim, Norway; ola.m.vagnildhaug@ntnu.no (O.M.V.); trude.r.balstad@ntnu.no (T.R.B.);; 2 Cancer Clinic, St. Olavs Hospital-Trondheim University Hospital, 7006 Trondheim, Norway; 3 Department of Clinical Medicine, Metabolism and Clinical Nutrition Research Group, UiT The Arctic University of Norway, 9019 Tromsø, Norway; 4 Department of Public Health and Nursing, NTNU-Norwegian University of Science and Technology, 7491 Trondheim, Norway; 5 Department of Nursing and Health Promotion, Faculty of Health Sciences, OsloMet-Oslo Metropolitan University, 0130 Oslo, Norway; 6 European Palliative Care Research Centre (PRC), Department of Oncology, Oslo University Hospital and Institute of Clinical Medicine, University of Oslo, 0424 Oslo, Norwaynaa@ous-hf.no (N.A.);; 7 Regional Advisory Unit for Palliative Care, Department of Oncology, Oslo University Hospital, University of Oslo, 0318 Oslo, Norway; 8 Department of Circulation and Medical Imaging, NTNU- Norwegian University of Science and Technology, 7491 Trondheim, Norway; 9 Department of Anesthesiology and Intensive Care Medicine, St. Olavs Hospital-Trondheim University Hospital, 7006 Trondheim, Norway

**Keywords:** cancer, cachexia, anorexia, appetite loss, metastases, inflammation, cytokines

## Abstract

Background: Inflammation is suggested to play a central role in cancer-related appetite loss, yet the available evidence remains limited. Methods: This study analyzed data from a prospective, longitudinal, multicenter study of patients with metastatic cancer followed for one year. The longitudinal associations between appetite loss and both clinical characteristics and inflammatory markers were examined. Logistic generalized equation model (GEE) was used. Results: Among 526 patients, 299 (57%) experienced appetite loss at baseline. During longitudinal observation appetite loss was associated with older age (OR 1.19, CI 1.01–1.39 as per 10-year years increase), poorer performance status (OR 0.85, CI 0.77–0.94), recent weight loss (OR 2.74, CI 2.11–3.56), and symptoms including constipation, nausea, fatigue, and lower emotional functioning. Corticosteroid use was associated with lower risk of appetite loss (OR 0.69, CI 0.53–0.90). Several inflammatory markers were also associated with appetite loss, including IL-8, IP-10, IL-6, CRP, eotaxin, albumin. Conclusions: Appetite loss in metastatic cancer is linked to clinical factors, symptom burden, and inflammatory markers. Optimizing symptom management and targeting inflammation might have a positive impact.

## Simple Summary

This study investigated 526 patients with advanced cancer followed for one year, to learn more about appetite loss and factors linked to it. More than half of the patients experienced reduced appetite. Appetite loss was linked to older age, poorer functional status, weight loss, fatigue, nausea, constipation and low mood. Appetite loss was also associated with signs of inflammation measured in blood. Patients receiving corticosteroids were less likely to report appetite loss. The findings supports a link between inflammation, symptom burden and appetite loss in advanced cancer, and may help guide further research aimed at improving the management of appetite loss.

## 1. Introduction

Appetite loss is burdensome and experienced by up to 80% of patients living with advanced cancer [1]. Understanding its diverse causes and contributing factors is crucial for developing future treatment strategies. Appetite loss in patients with advanced cancer may result from acute infections or injuries, treatment-related side effects or underlying conditions and symptoms such as depression or constipation [2]. Appetite loss can also be a component of cachexia, a systemic disorder with complex pathophysiology, where inflammation caused by tumor–host interactions seems to be at its core [3,4].

The associations between appetite loss and inflammatory cytokines have been studied in patients with advanced cancer [5,6]. A few cross-sectional studies have reported a significant association between elevated interleukin-6 (IL-6) levels and appetite loss [5,7,8]. Other papers describe associations with IL-8, IL-1β and tumor necrosis factor (TNF) [5,9]. To our knowledge, only one study previously explored whether changes in cytokines affect appetite in humans [10]. However, animal studies have demonstrated that the injection of cytokines or the pharmacological disruption of cytokines prospectively affect appetite [2,11]. Longitudinal human studies exploring the role of inflammatory cytokines in appetite loss have consequently been advocated [6].

Appetite loss in advanced cancer patients is understudied [4,12]. Loss of appetite not only contributes to reduced nutritional intake, aggravating muscle and fat wasting but also represents a burdensome symptom causing profound distress for both patients and carers [13]. Therefore, it is imperative to explore the relationship between appetite loss and associated factors during the disease course. The primary objective of this study was to identify and longitudinally observe clinical factors and inflammatory markers associated with appetite loss in patients with advanced cancer.

## 2. Materials and Methods

### 2.1. Patient Population and Study Design

Between December 2013 and December 2017, 574 patients from seven oncological study centers across Europe (Norway, Italy, Spain and the United Kingdom) were included in the Palliative Radiotherapy and Inflammation Study (PRAIS). This was a prospective observational study exploring the associations between inflammation and pain, cachexia, and depression in patients with cancer-induced bone pain [14]. Eligible patients met the following key criteria: ≥18 years of age with a verified cancer diagnosis and about to undergo radiotherapy with palliative intent for painful bone metastases. Participants with available symptom registrations and inflammatory cytokines at baseline were included in the present analysis.

### 2.2. Data Collection

Patients were assessed at baseline (before radiotherapy), with follow-up measures at 3, 8, 16, 24 and 52 weeks after radiotherapy, or until study withdrawal or death [14]. The clinical factors selected were based on known relationship with cachexia or appetite loss. [1,3,4,15]. The following data were used: Patient demographics, Karnofsky performance status, the characteristics of the cancer disease (cancer diagnosis, distribution of metastases, years since cancer diagnosis), systemic cancer treatment (including chemotherapy, hormonal therapy or other anticancer treatments), the use of opioids, corticosteroids, antiemetics and antibiotics (yes/no). Patient-reported outcomes included weight loss within two weeks from the Patient-Generated Subjective Global Assessment Short Form (PG-SGA SF) [16] and symptom scales for appetite loss, constipation, nausea, pain, fatigue and emotional functioning from the Treatment of Cancer Quality of Life Questionnaire (EORTC-QLQ) C15-PAL [17]. Items from the EORTC-QLQ-C15-PAL were summarized and linearly transformed into symptom scales ranging from 0–100 according to the EORTC-QLQ guidelines [17]. As the symptom scale for appetite loss only contains one item (appetite loss past week), this item was transformed to the dichotomous variable (yes/no) including appetite loss of any severity (a little, quite a bit and very much).

Blood samples were obtained at baseline and at follow-up visits. CRP (mg/L) and albumin (g/L) were analyzed locally [14]. A preplanned and detailed set of inflammatory markers was analyzed centrally with a multiplex cytokine assay (Bio-Plex Pro™ Human Cytokine Plex-27 Assay, Bio-Rad Laboratories, Hercules, CA, USA). The analytical methods are described in more detail elsewhere [18]. Inflammatory markers with low or nondetectable levels in more than 20% of the patients were not included in further analyses. A final sample of 13 inflammatory cytokines are presented in the current analysis: TNF, IL-1ra, IL-8, IL-9, IL-6, IL-7, IL-17a, granulocyte colony-stimulating factor (G-CSF), interferon gamma-induced protein-10 (IP-10), eotaxin, macrophage inflammatory protein-1 β (MIP-1 β), basic fibroblast growth factor basic FGF) and monocyte chemoattractant protein-1 (MCP-1). We aimed to report both inflammatory markers with a known association with appetite loss (IL-6, IL-8, and TNF) and inflammatory markers with an uncertain association with appetite loss [5]. Inflammatory cytokines are reported as pg/mL and binary logarithmic (log_2_) transformed to obtain a normal distribution.

### 2.3. Statistical Considerations

Baseline demographics are presented as absolute numbers (N) with corresponding percentages (%) and means with standard deviations (SD). The number of patients who experienced appetite loss at each follow-up time point is illustrated graphically. Longitudinal data analyses were conducted to identify factors associated with appetite loss at each specific time point during the study. Fixed variables included age, sex, cancer diagnosis, years since cancer diagnosis and systemic cancer treatment prior to enrollment. Time-varying variables included Karnofsky performance status, metastatic status, symptom-directed medications (opioids, corticosteroids, antiemetics and antibiotics), weight loss within the past two weeks, patient reported symptoms (pain, emotional functioning, fatigue, nausea, and constipation) and blood samples for inflammatory markers. For the clinical variables, multivariate associations were explored. All relevant variables were included in the model without performing any backward or forward selection. For the inflammatory markers, univariate associations were explored to avoid multicollinearity. All analyses were performed with a logistic generalized equation (GEE) model to include measures at all time points and adjust for repeated measures [19]. An exchangeable covariance structure was selected on the basis of the expectancy of the data output, the quasi likelihood independence model criterion (QIC), and the model that included the most observations [20]. To improve interpretability of model estimates, age, Karnofsky performance status and patient reported symptom scores (EORTC-QLQ-C15-PAL) were scaled into 10-unit increasements. Regression diagnostics were performed for all analyses, and potential interactions were investigated. To assess the robustness of the findings to attrition at later follow-up assessments, sensitivity analyses were conducted by repeating the models after excluding observations from week 24 and week 52. To minimize the risk of Type II errors, no adjustment for multiple comparison was performed. Consequently, the reported *p*-values should be interpreted with caution. For descriptive purposes, predictive probabilities were estimated from the clinical model of appetite loss. Analyses were performed with STATA version 18.0 (Stata Corporation LP; College Station, TX, USA).

### 2.4. Ethical Considerations

The study was approved by the Regional Committee for Medical and Health Research Ethics (2013/1126/REK midt) as well as medical research ethics committees in each country. All patients provided written informed consent before inclusion.

## 3. Results

**Figure 1:**
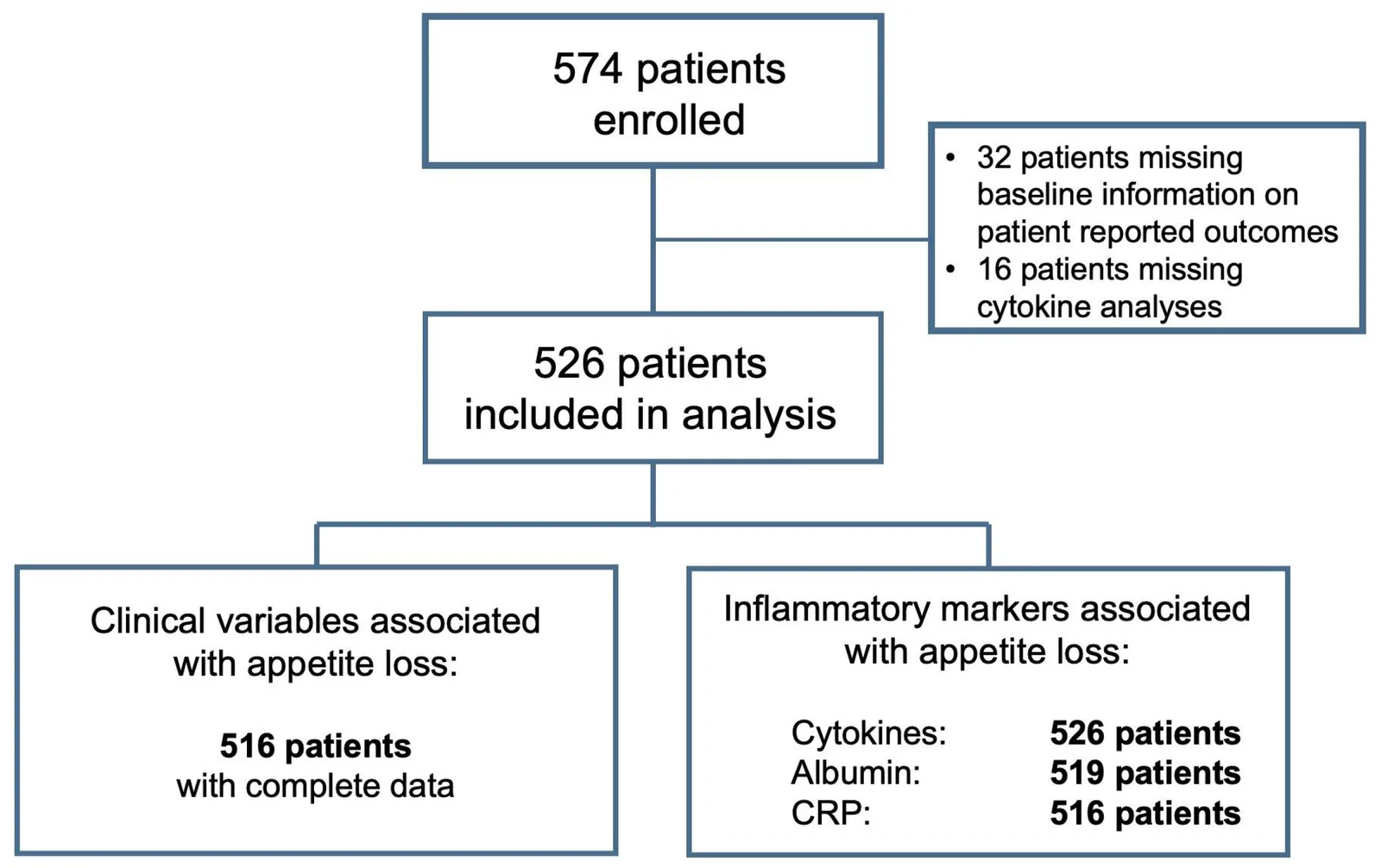
Flowchart of patients included in the analyses.

Among 574 patients enrolled in PRAIS, the 526 patients with available baseline measurements of inflammatory cytokines and self-reported appetite were analyzed (Figure 1). Mean age was 66 years (SD 10.6), and 328 (62%) of the included patients were male. Mean Karnofsky performance status was 72.3 (SD 12.3). Prostate (25%), breast (18%), lung (18%) and gastrointestinal (17%) cancer were most common. All included patients had bone metastases, 29% had lung metastases, and 28% had liver metastases (Table 1).

**Figure 2:**
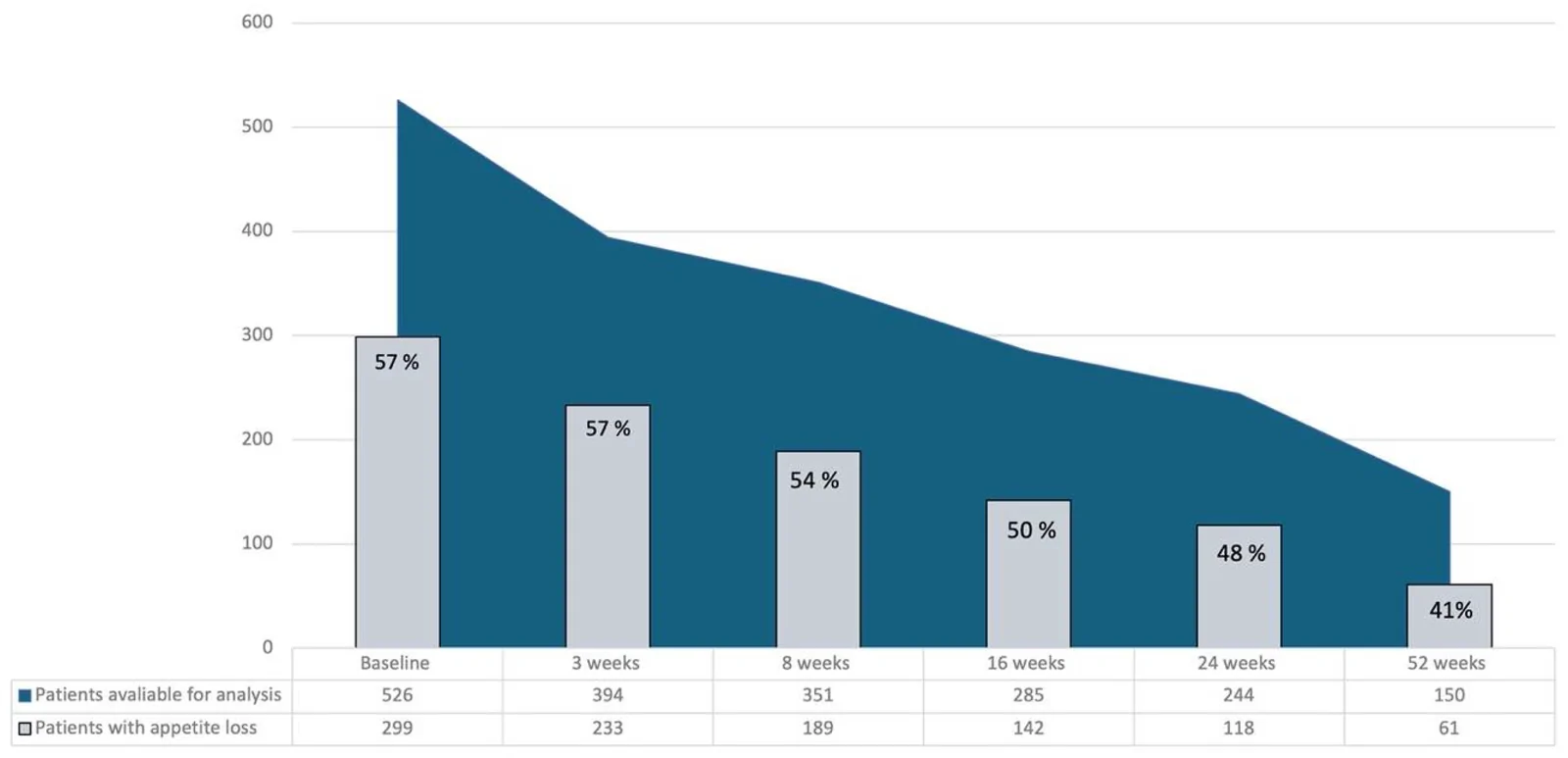
Appetite loss in patients available for analysis at each follow-up visit.

Appetite loss was reported by 299 patients (57%) at baseline, and little individual variation in appetite loss were observed throughout the observation period. The number of patients available for follow-up decreased over time, as did the proportion of patients reporting appetite loss (Figure 2). Missing follow-up data were partly attributable to patient mortality during the study period. Twenty-eight patients died before the 3-week follow-up, while the cumulative numbers of deaths were 93, 150 and 188 at weeks 8, 16 and 24 respectively. By the 1-year follow-up, 332 patients had died. Most remaining missing observations were due to loss to follow-up.

### 3.1. Clinical Characteristics and Symptoms Associated with Appetite Loss

**Figure 3:**
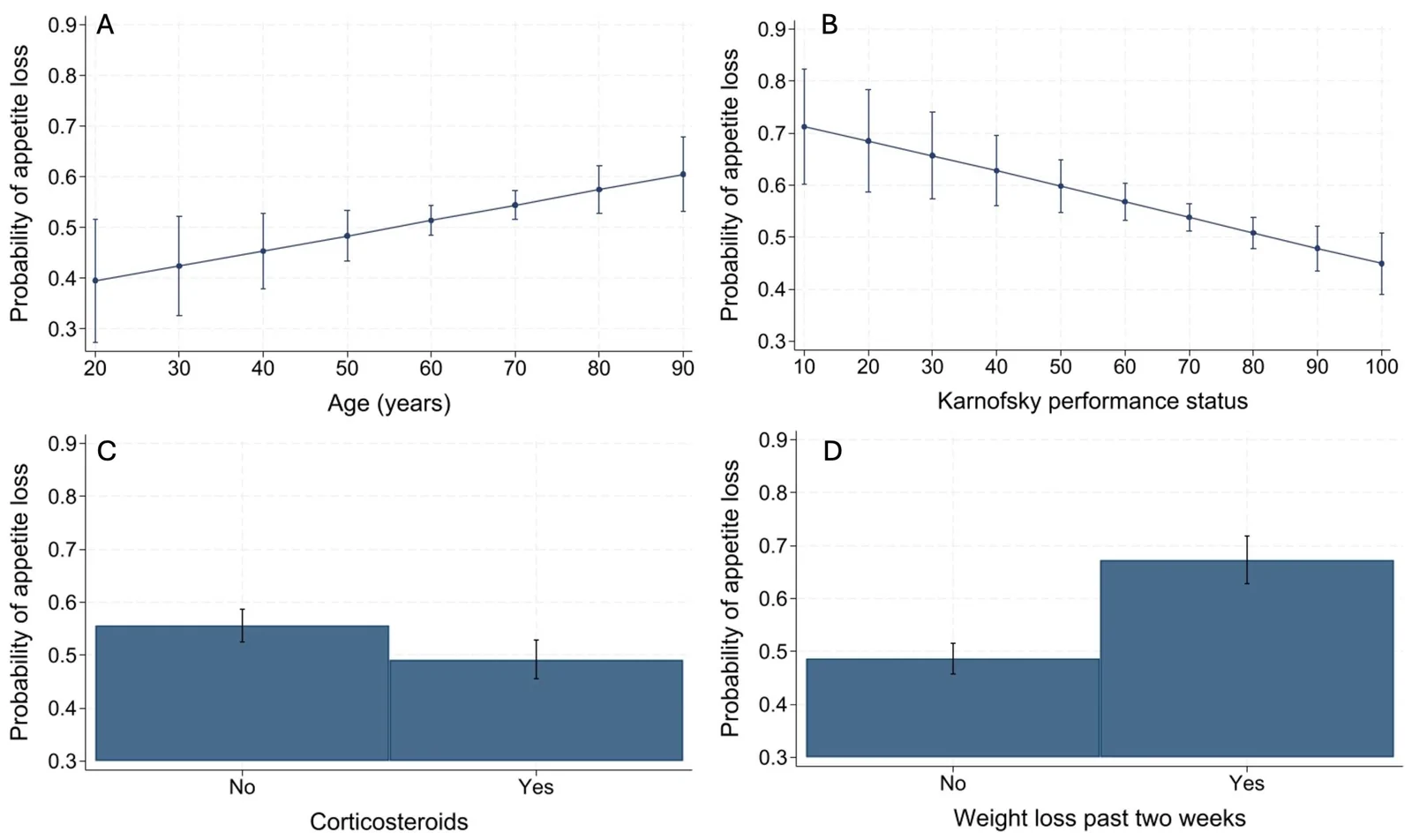
The predicted probability of appetite loss by clinical variables. On the *x*-axis, the following clinical variables were used: (**A**) age in years, (**B**) Karnofsky performance status, (**C**) use of corticosteroids and (**D**) weight loss within two weeks. The *y*-axis shows the probability of anorexia. The 95% confidence interval is presented with vertical lines. The predicted probabilities are derived from longitudinal analysis presented in Table 2.

**Figure 4:**
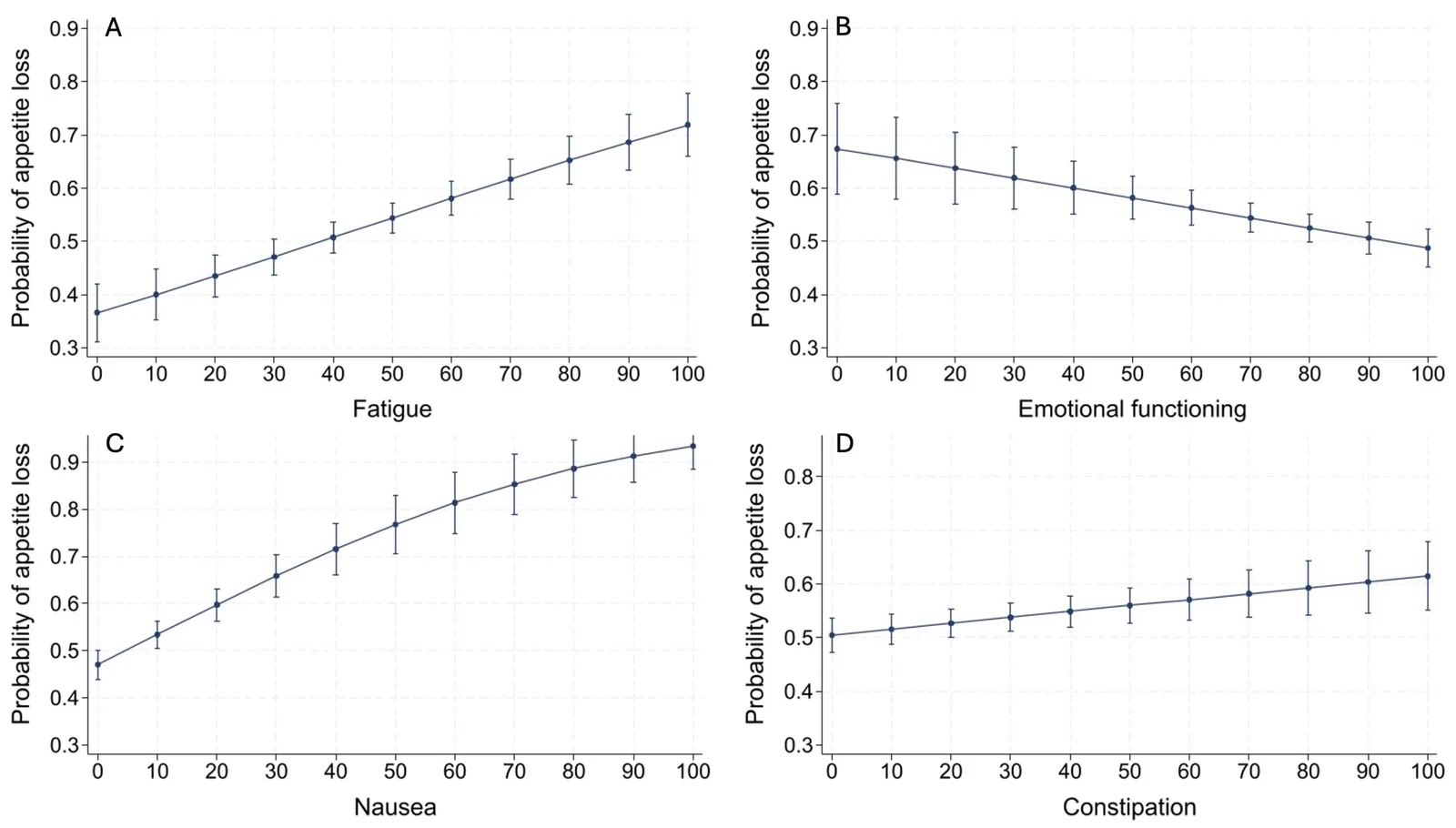
The predicted probability of appetite loss by patient reported symptoms. On the *x*-axis, symptom scores for (**A**) fatigue, (**B**) emotional functioning, (**C**) nausea and (**D**) constipation. The *y*-axis shows the probability of anorexia. The 95% confidence interval is presented with vertical lines. The predicted probabilities are derived from longitudinal analysis presented in Table 2.

Older age (OR 1.19, CI 1.01–1.39 as per 10-years increase), lower Karnofsky performance status (OR 0.85, CI 0.77–0.94 as per 10 points increase), and weight loss within two weeks (OR 2.74, CI 2.11–3.56) were significantly associated with appetite loss during the longitudinal observational period. Patients receiving corticosteroids experienced less frequent appetite loss (OR 0.69, CI 0.53–0.90). No significant differences in prevalence of appetite loss were observed across cancer diagnoses. Appetite loss was associated with several patient-reported symptoms. Patients with appetite loss reported more severe constipation (OR 1.06, CI 1.02–1.11), nausea (OR 1.39 CI 1.27–1.51) and fatigue (OR 1.21, CI 1.14–1.28) as well as lower emotional functioning (OR 0.90, CI 0.85–0.96) (Table 2). No relevant interactions among the clinical variables were identified. The results remained largely unchanged in sensitivity analyses excluding observations from week 24 and week 52 (Table S1). The predictive probabilities for appetite loss and the significant clinical variables are presented in Figure 3 and Figure 4.

### 3.2. Inflammatory Markers Associated with Appetite Loss

Appetite loss was significantly associated with higher levels of IL-8 (OR 1.3, CI 1.17–1.45), IP-10 (OR 1.19, CI 1.08–1.31), IL-6 (OR 1.2, CI 1.12–1.27) and CRP (OR 1.01, CI 1.0–1.01) and lower levels of eotaxin (OR 0.82 CI 0.70–0.95) and albumin (OR 0.93 CI 0.91–0.95) (Table 3). The results remained stable in sensitivity analyses excluding observations from week 24 and week 52 (Table S2).

## 4. Discussion

The study identified several clinical factors and inflammatory markers associated with appetite loss in patients with metastatic cancer. Appetite loss was associated with older age, reduced performance status, recent weight loss, constipation, nausea, fatigue and lower emotional functioning. Corticosteroid use was associated with lower odds of appetite loss. Several inflammatory markers were also associated with appetite loss, suggesting a link between systemic inflammation and appetite.

Appetite loss is common and clinically important in patients with advanced cancer, particularly in older individuals at increased risk of undernutrition [21]. Our findings confirm this observation. Although the effect of age on appetite loss was modest, the odds of appetite loss increased by approximately 19% for every 10-year increase in age. This association may reflect age-related physiological changes, as well as polypharmacy and comorbidities. The observations emphasize the importance of systematic screening for appetite loss in older patients. Early identification and intervention may help prevent undernutrition, improve treatment tolerance, and potentially enhance survival outcomes [22].

Consistent with previous studies, appetite loss was strongly associated with reduced performance status and weight loss [23]. Whether appetite loss contributes directly to functional decline and weight loss, or whether these factors represent parallel manifestations of cancer cachexia remain uncertain. These findings should therefore be interpreted as associations rather than causal effects. The lack of association with cancer type suggest that appetite loss is a more universal symptom in advanced cancer, potentially less influenced on primary tumor characteristics than cancer cachexia [12,23,24].

Symptom burden appeared closely linked to appetite loss, with nausea showing the strongest association, underscoring the importance of optimal symptom control [25]. Although antiemetic use was not associated with appetite loss, interpretation of this finding is limited by the lack of data on treatment type and dosage. Lower emotional functioning was also associated with appetite loss, supporting a close relationship between phycological well-being and appetite loss in cancer patients. Given that depression may influence appetite, these findings highlight the importance of integrated approaches addressing both physical and psychological dimensions [26].

The findings might support a multimodal approach to appetite loss [27,28]. Olanzapine has emerged as a promising option because of its potential effects on both appetite, nausea and psychological symptoms [29]. Corticosteroids have also long been used to manage cancer-related appetite loss, but evidence of their efficacy is mixed [28,30,31]. While corticosteroid use was associated with lower odds of appetite loss in the present study, this finding should be interpreted with caution. Data on corticosteroid type, dose, duration and indication were unavailable, and residual confounding cannot be excluded.

Systemic inflammations is believed to be an important contributor to cachexia development [3]. Previous cross-sectional studies have reported associations between appetite loss, inflammatory markers and other cancer-related symptoms [4,5,6,8,32]. Extending these findings, we observed longitudinal associations between appetite loss and both IL-8 and IL-6, which is consistent with a potential role of inflammatory pathways in appetite regulation. This is supported by recent evidence that IL-6 inhibitors might mitigate appetite loss [33]. Furthermore, CRP, a downstream marker of IL-6 activity, was also associated with appetite loss in our study and previous reports [34], providing further support for an association between systemic inflammation and appetite loss. Albumin is a negative acute-phase reactant and may decrease during systemic inflammation independent of nutritional intake [35]. We observed significant lower albumin levels in patient with appetite loss, like one previous cross-sectional study [36]. Given the widespread availability in routine clinical practice, albumin may be a useful biomarker for identifying patients at increased risk of appetite loss, although this requires further validation.

We also identified potentially novel and significant associations with inflammatory markers that have not previously been linked to appetite or cancer cachexia. Specifically, patients with appetite loss presented elevated IP-10 and reduced eotaxin levels. IP-10 (CXCL10) is a chemokine involved in various inflammatory processes, including responses to infection, autoimmunity, and cancer progression, suggesting a potential role in the inflammatory mechanisms underlying cancer-related appetite loss [37]. Although its association with appetite loss in cancer remains unclear, parallels can be drawn to the systemic inflammatory response observed in severe infections, such as COVID-19, in a noncancer population. In this context, IP-10 has been identified as a key inflammatory mediator contributing to inflammation-induced cachexia [38]. Eotaxin is a small cytokine that acts as a chemokine and primarily attracts eosinophils. While a high level of eotaxin is associated with allergic diseases, it has also been linked to obesity and is known to decrease with weight loss [39,40]. Moreover, emerging evidence suggests that eotaxin is related to leptin, a hormone important for regulating food intake, indicating a potential role for eotaxin in appetite regulation [41]. However, these findings should be interpreted cautiously, as multiple inflammatory biomarkers were evaluated without correction for multiple comparison. The observed associations should therefore be considered exploratory. Some inflammatory markers of potential interest, such as TNF, were not significantly associated with appetite loss in the current analysis [6,7].

Finally, although this was a longitudinal study, the observed relationships should be interpreted as associations rather than causal effects. Several of the examined variables may reflect common underlying mechanisms related to cancer progression and cachexia, making it difficult to disentangle their individual contributions to appetite loss.

### Strengths and Limitations

Strengths of this study include its large sample size, availability of blood samples for inflammatory marker analysis, and longitudinal design enabling observation of symptom trajectories. Clinical and biological variables were predefined and published before initiating the analyses, based on known associations with appetite loss and cachexia at the time planning for the study [14]. The baseline prevalence of appetite loss (57%) was consistent with other palliative populations [15]. Limitations include substantial loss to follow-up, including attrition due to death, which is anticipated in research involving a metastatic cancer population. Nutritional interventions were not systematically recorded, and potential center effects were not examined. Changes in anticancer treatment during follow-up were not accounted for and may have influenced appetite and inflammatory status. Furthermore, reliance on a single-item assessment of appetite loss may have reduced measurement precision and may not fully capture the multidimensional nature of appetite disturbances. Systemic inflammation may also have been influenced by infections, medications, corticosteroid use, and radiotherapy, which could affect levels of inflammatory markers. Appetite may also have been influenced by other clinical variables or inflammatory markers not measured in the current study. Finally, as multiple inflammatory markers and clinical variables were evaluated, the possibility of type I error should be considered when interpreting individual associations.

## 5. Conclusions

Appetite loss is a common and persistent symptom in metastatic cancer. Clinicians should pay particular attention to older patients, those with poor performance status, and those experiencing weight loss. Corticosteroid use was associated with lower odds of appetite loss. Lower emotional functioning, fatigue, nausea, and constipation increased the risk of appetite loss and represent potential treatment targets. Associations with inflammatory markers, including IL-8, IP-10, IL-6, CRP, eotaxin and albumin, suggest a link between inflammation and appetite loss. Further mechanistic and interventional studies are needed to determine whether these pathways represent potential therapeutic targets.

## Figures and Tables

**Table 1:** Patient demographics and symptoms.

Characteristics	Sample	%
Age (mean, SD)	66.0 (10.6)	
Gender		
Male	328	62.3
Female	198	37.6
Karnofsky Performance status (mean, SD)	72.3 (12.3)	
Cancer diagnosis		
Prostate	134	25.5
Breast	96	18.3
Lung	96	18.3
Gastrointestinal	90	17.1
Urological	55	10.5
Other	55	10.5
Liver metastases		
Yes	147	27.9
No	379	72.1
Lung metastases		
Yes	154	29.3
No	372	70.7
Brain metastases		
Yes	32	6.1
No	494	93.9
Years since cancer diagnosis (mean, SD)	4.1 (4.8)	
Systemic cancer treatment ^2^		
Yes	334	63.5
No	192	36.5
Opioids		
Yes	423	80.4
No	102	19.4
Missing	1	0.2
Corticosteroids		
Yes	240	45.6
No	283	53.8
Missing	3	0.6
Antiemetics		
Yes	121	23
No	397	75.5
Missing	8	1.5
Antibiotics		
Yes	27	5.1
No	491	93.4
Missing	8	1.5
Weight loss past two weeks		
Yes	148	28.1
No	363	69.0
Missing	15	2.9
Appetite loss		
Yes	299	56.8
No	227	43.2
Cancer-related symptom score ^1^ (mean, SD)		
Pain	64.4 (24.7)	
Emotional functioning	70.5 (23.1)	
Fatigue	52.0 (25.6)	
Nausea	14.2 (22.7)	
Constipation	31.9 (35.9)	

^1^ Cancer-related symptoms form the Treatment of Cancer Quality of Life Questionnaire (EORTC-QLQ) C15-PAL (range 0–100); ^2^ systemic cancer treatment (chemotherapy, hormonal therapy or other anticancer therapies) within 6 weeks prior to enrollment.

**Table 2:** Longitudinal association of appetite loss with clinical characteristics and symptoms (N. 516).

	OR	95% CI	*p*-Value
Age ^1^	1.19	1.01–1.39	**0.035**
Female sex	1.13	0.74–1.72	0.576
Karnofsky Performance status ^2^	0.85	0.77–0.94	**0.002**
Cancer diagnosis			
Prostate	-	-	ref. category
Breast	1.03	0.54–1.96	0.928
Lung	1.52	0.90–2.59	0.117
Gastrointestinal	1.14	0.63–2.06	0.672
Urological	0.92	0.51–1.67	0.796
Other	1.57	0.84–2.94	0.159
Metastases			
Liver	1.35	0.96–1.89	0.083
Lung	1.19	0.85–1.68	0.300
Brain	0.72	0.42–1.26	0.251
Years since cancer diagnosis	0.99	0.95–1.02	0.408
Systemic cancer treatment ^3^	0.88	0.62–1.24	0.467
Opioids	1.33	0.99–1.80	0.060
Corticosteroids	0.69	0.53–0.90	**0.006**
Antiemetics	1.04	0.76–1.42	0.815
Antibiotics	1.06	0.65–1.73	0.821
Weight loss past two weeks	2.74	2.11–3.56	**<0.001**
Cancer-related symptoms ^4^			
Pain	1.01	0.96–1.06	0.680
Emotional functioning	0.90	0.85–0.96	**0.001**
Fatigue	1.21	1.14–1.28	**<0.001**
Nausea	1.39	1.27–1.51	**<0.001**
Constipation	1.06	1.02–1.11	**0.005**
Constant	0.41	0.07–2.25	0.304

^1^ Age as per 10 years increase. ^2^ Karnofsky performance status (range 0–100), as per 10 points increase. ^3^ Systemic cancer treatment (chemotherapy, hormonal therapy or other anticancer therapies) within 6 weeks prior to enrollment. ^4^ Cancer-related symptoms from the Treatment of Cancer Quality of Life Questionnaire (EORTC-QLQ) C15-PAL (range 0–100), as per 10 points increase. N = The number for unique patients with complete data contributing to the longitudinal model. *p*-values < 0.05 are shown in bold.

**Table 3:** Longitudinal associations between appetite loss and inflammatory markers.

	OR	95% CI	*p*-Value
TNF	1.11	0.98–1.26	0.110
IL1ra	1.01	0.99–1.04	0.382
IL-8	1.30	1.17–1.45	**<0.001**
IL-9	0.98	0.87–1.11	0.806
IP-10	1.19	1.08–1.31	**<0.001**
Eotaxin	0.82	0.70–0.95	**0.009**
MIP-1β	1.01	0.85–1.20	0.912
G-CSF	1.12	0.98–1.28	0.076
IL-6	1.20	1.12–1.27	**<0.001**
IL-7	1.04	0.96–1.12	0.305
IL-17A	0.88	0.74–1.05	0.166
MCP-1	0.98	0.89–1.09	0.759
Basic FGF	1.01	0.98–1.04	0.594
CRP	1.01	1.00–1.01	**<0.001**
Albumin	0.93	0.91–0.95	**<0.001**

Abbreviations: TNF = tumor necrosis factor, IL = interleukin, IP-10 = interferon gamma-induced protein-10, MIP-1β = macrophage inflammatory protein 1 beta, G-CSF = granulocyte colony-stimulating factor, MCP-1 = monocyte chemoattractant proteine-1, Basic FGF = basic fibroblast growth factor, CRP = c-reactive protein. Number of patients in analysis is 526 for all inflammatory markers except albumin (519) and CRP (516). *p*-values < 0.05 are shown in bold.

## Data Availability

The data that support the findings of this study are not openly available for reasons of sensitivity and are available from the corresponding author upon reasonable request.

## Supplementary Materials

The following supporting information can be downloaded at https://www.mdpi.com/article/10.3390/cancers18203407/s1: Table S1. Sensitivity analyses for the longitudinal association of appetite loss with clinical characteristics and symptoms, restricted to observations within the first 16 weeks; Table S2. Sensitivity analyses on the longitudinal associations between appetite loss and inflammatory markers restricted to observations within the first 16 weeks.
