# Supplementary material for: Appetite Loss in Metastatic Cancer: Insights from Clinical and Inflammatory Markers Observed Longitudinally
Source: Cancers (Basel). 2026 Oct 21;18(20):3407. doi: 10.3390/cancers18203407 (PMC13702586; doi:10.3390/cancers18203407)
Supplement: Supplementary file 1 [file cancers-18-03407-s001.zip › cancers-4614090-supplementary.pdf]

Supplementary Materials

Table S1. Sensitivity analyses for the longitudinal association of appetite loss with clinical characteristics and symptoms, restricted to observations within the first 16 weeks.

	**OR**	**95 % CI**	***p*-value**
Age	1.18	1.00-1.40	**0.043**
Female sex	1.06	0.68-1.63	0.786
Karnofsky Performance status	0.80	0.72-0.94	**<0.001**
Cancer diagnosis			
Prostate	-	-	ref. category
Breast	1.05	0.54-2.05	0.880
Lung	1.33	0.77-2.29	0.303
Gastrointestinal	1.10	0.63-2.06	0.754
Urological	0.92	0.60-2.04	0.796
Other	1.55	0.81-2.98	0.183
Metastases			
Liver	1.41	0.98-2.03	0.063
Lung	1.21	0.78-1.61	0.533
Brain	0.74	0.42-1.32	0.318
Years since cancer diagnosis	0.99	0.95-1.02	0.408
Systemic cancer treatment[2]	0.88	0.62-1.24	0.467
Opioids	1.29	0.92-1.82	0.130
Corticosteroids	0.60	0.45-0.80	**0.001**
Antiemetics	1.10	0.77-1.56	0.600
Antibiotics	1.11	0.64-1.92	0.704
Weight loss past two weeks	2.56	1.90-3.43	**<0.001**
Cancer related symptoms[1]			
Pain	1.0	0.95-1.05	0.989
Emotional functioning	0.89	0.83-0.95	**<0.001**
Fatigue	1.17	1.10-1.24	**<0.001**
Nausea	1.45	1.30-1.61	**<0.001**
Constipation	1.05	1.0-1.10	**0.026**
constant	0.41	0.07-2.25	0.304

[1] Cancer related symptoms form the Treatment of Cancer Quality of Life Questionnaire (EORTC-QLQ) C15-PAL (range 0-100)
[2] Systemic cancer treatment (chemotherapy, hormonal therapy or other anticancer therapies) within 6 weeks prior to enrollment
N = The number for unique patients with complete data contributing to the longitudinal model.

Table S2 . Sensitivity analyses on the longitudinal associations between appetite loss and inflammatory markers restricted to observations within the first 16 weeks.

	**OR**	**95 % CI**	***p*-value**
TNF	1.11	0.97-1.27	0.120
IL1ra	1.01	0.98-1.04	0.485
IL-8	1.31	1.16-1.47	**<0.001**
IL-9	0.99	0.87-1.13	0.882
IP-10	1.17	1.06-1.29	**0.003**
Eotaxin	0.82	0.69-0.96	**0.014**
MIP1b	1.02	0.85-1.22	0.867
G-CSF	1.12	0.97-1.28	0.118
IL-6	1.19	1.12-1.28	**<0.001**
IL-7	1.04	0.96-1.14	0.309
IL-17A	0.88	0.73-1.05	0.157
MCP-1	0.99	0.89-1.10	0.845
Basic FGF	1.00	0.97-1.04	0.899
CRP	1.01	1.00-1.01	**<0.001**
Albumin	0.93	0.91-0.96	**<0.001**

Abbreviations: TNF = tumor necrosis factor, IL = interleukin, IP-10 = interferon gamma-induced protein-10, MIP-1β = macrophage inflammatory protein 1 beta, G-CSF = granulocyte colony-stimulating factor, MCP-1 = monocyte chemoattractant proteine-1, Basic FGF = basic fibroblast growth factor, CRP = c-reactive protein. Number of patients in analysis is 526 for all inflammatory markers except albumin (519) and CRP (516).
